# Supplementary figures and images for: Survival and growth of saprotrophic and mycorrhizal fungi in recalcitrant amine, amide and ammonium containing media
Source: PLoS One. 2021 Sep 1;16(9):e0244910. doi: 10.1371/journal.pone.0244910 (PMC8409640; doi:10.1371/journal.pone.0244910)

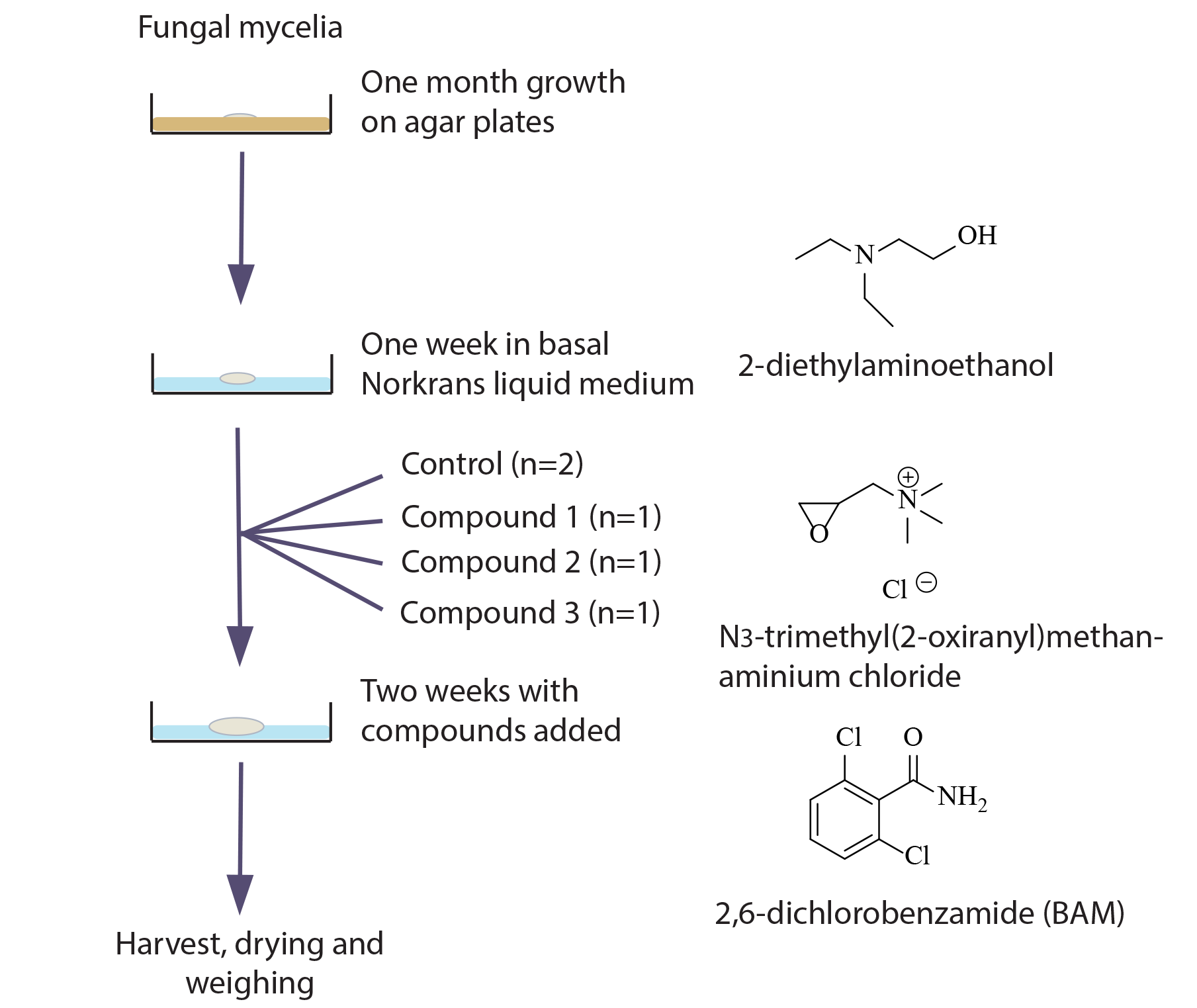

Supplement: S1 Fig — A total of 48 fungal isolates where grown in Petri dishes for a total of three weeks in liquid growth media containing individual recalcitrant compounds. Concentrations for the N-containing compounds were 1 g L-1, 10 g L-1, and 20 g L-1 for 2-diethylaminoethanol and N3-Trimethyl(2-oxiranyl)methanaminium chloride, and for 2,6-dichlorobenzamide (BAM) 1 μL, 1 mL, and 2 mL were added from a saturated solution. No substance was added to the growth controls. The recalcitrant N-containing compounds are depicted to the right. (TIF) [file pone.0244910.s001.tif]

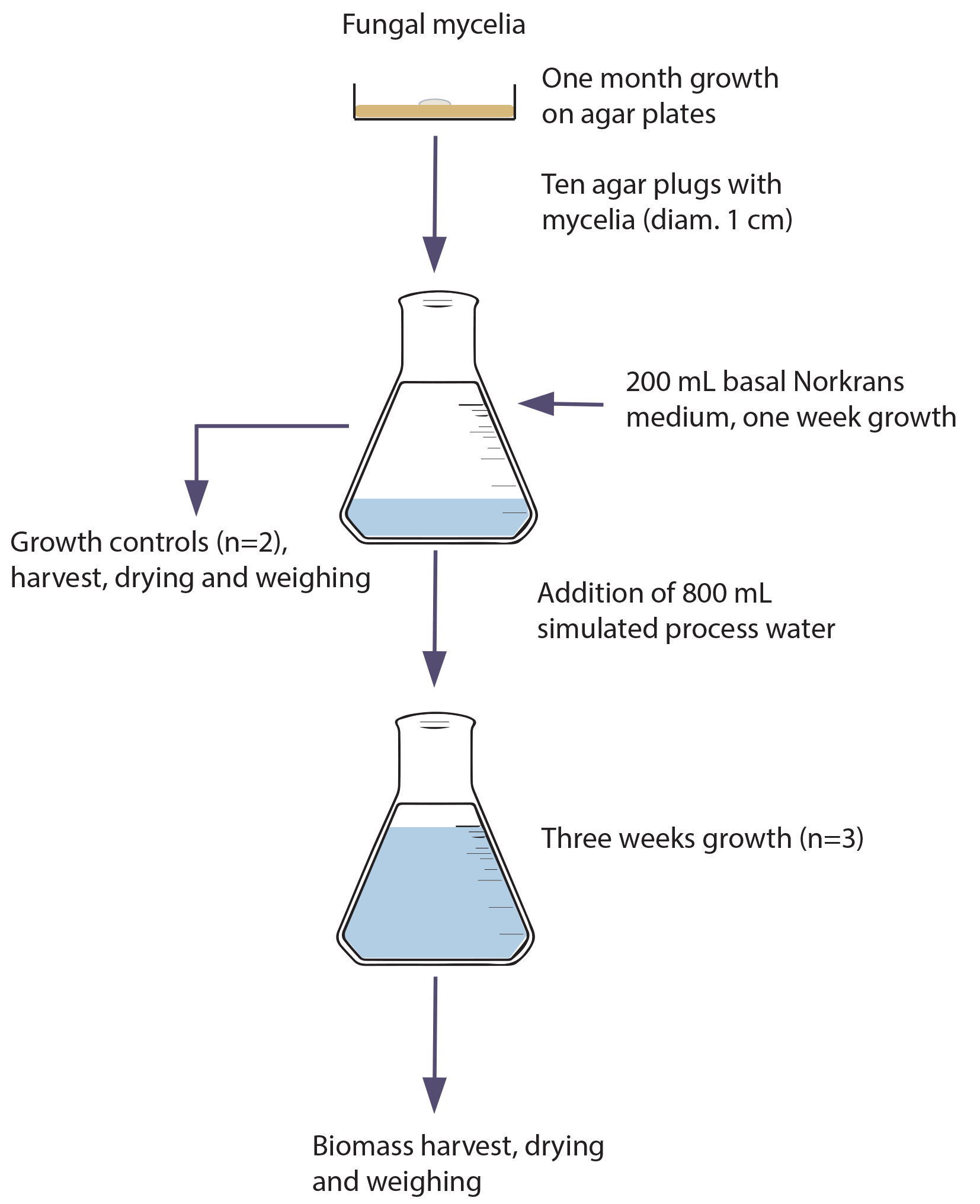

Supplement: S2 Fig — Three mycorrhizal fungal species (Hygrophorus camarophyllus, Rhizoscyphus ericae, and Laccaria laccata) were grown for a total of four weeks in Erlenmeyer flasks containing a recalcitrant amine/amide mixture. For composition of mixture see Table 1. (TIF) [file pone.0244910.s002.tif]

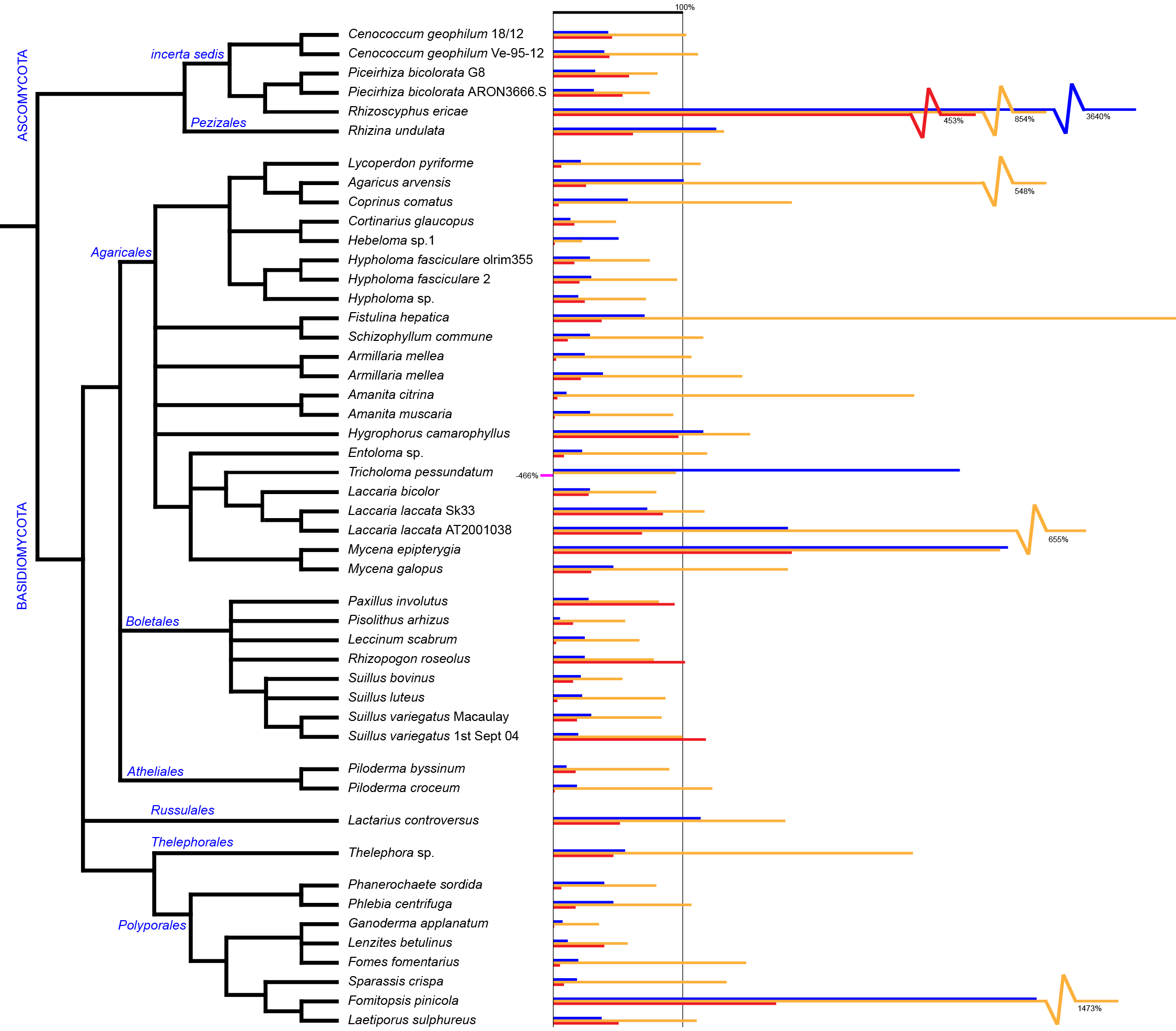

Supplement: S3 Fig — The growth responses represent the isolates (% of controls) containing the three highest N-containing compounds’ concentrations. Concentrations corresponded to 20 g L-1 for 2-diethylaminoethanol (blue bars) and N3-Trimethyl(2-oxiranyl)methanaminium chloride (orange bars), and addition of 2 mL saturated 2,6-dichlorobenzamide (BAM) solution (red bars). Values over 100% means that fungi grew better with the amines present. The red branches are ECM fungi, the blue saprotrophs and the green ERM mycorrhiza. The diagram was cut at 500%, missing values and negative values were set to 0%. (TIF) [file pone.0244910.s003.tif]

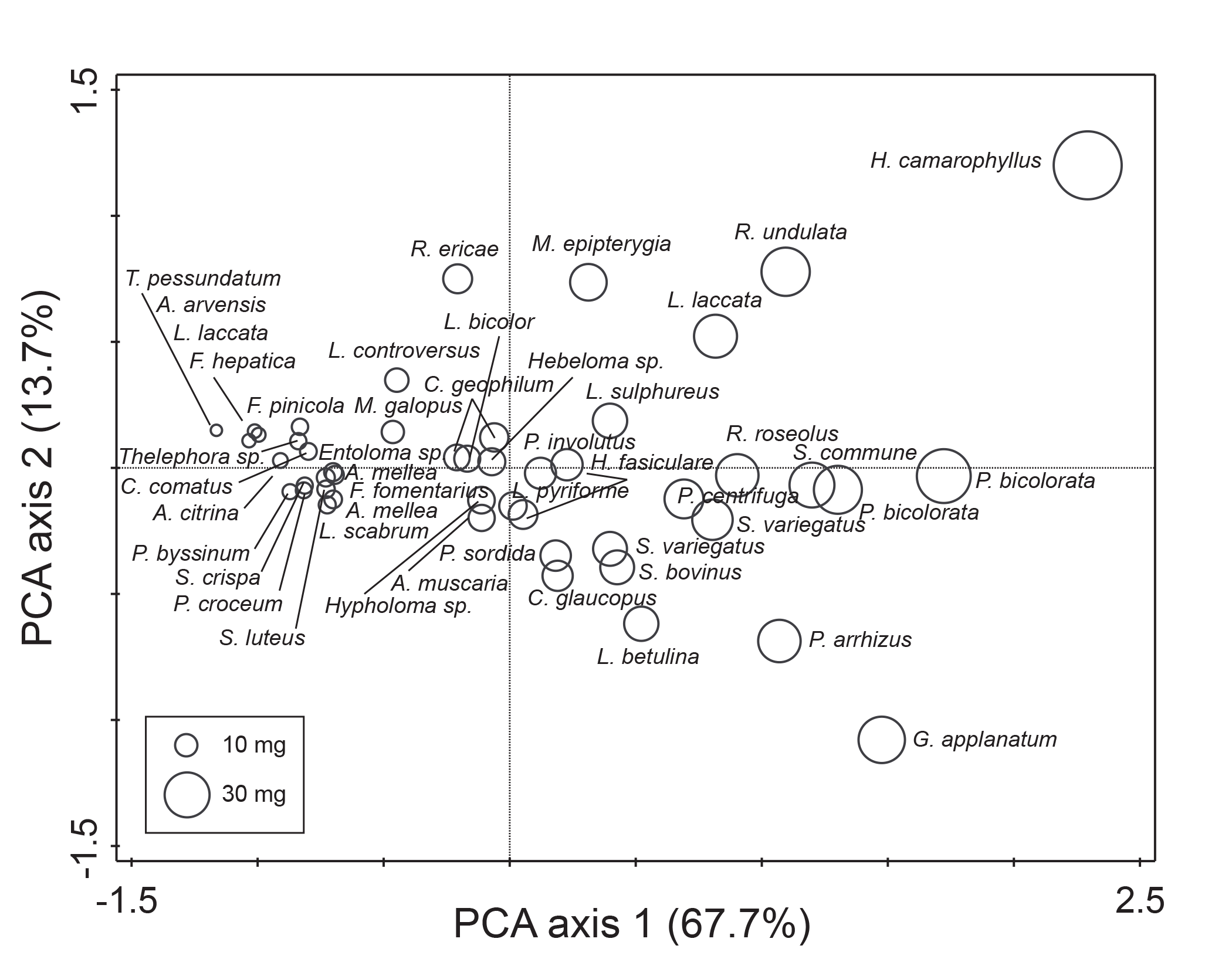

Supplement: S4 Fig — 48 fungal isolates when grown for three weeks in control treatment with nutrient solution and amine treatments (2-diethylaminoethanol [2-diet], N3-Trimethyl(2-oxiranyl)methanaminium chloride [gly] and 2,6-dichlorobenzamide [BAM]) at three different concentrations, respectively. For 2-diethylaminoethanol and N3-Trimethyl(2-oxiranyl)methanaminium chloride concentrations were 1, 10, and 20 g L-1. For BAM 1μL, 1 mL, and 2 mL of a saturated solution was added. Species differences are visualized by a sample plot with circle size depicting the average biomass response across all treatments. The first three axes together explained 87.5% of the total variation (84181.4). (TIF) [file pone.0244910.s004.tif]
