# Supplementary material for: Survival and growth of saprotrophic and mycorrhizal fungi in recalcitrant amine, amide and ammonium containing media
Source: PLoS One. 2021 Sep 1;16(9):e0244910. doi: 10.1371/journal.pone.0244910 (PMC8409640; doi:10.1371/journal.pone.0244910)
Supplement: Attachment — Submitted filename: Recommendations.docx [file pone.0244910.s009.docx]

Although authors has improved the manuscript markedly but still I have some queries and doubt regarding my earlier comments.

1. What is the history of sampling site? I mean fungal species isolated from any waste water disposal site would be more adaptable for degrading the recalcitrant compound compared to fungi isolated from healthy site.
2. Introduction needs to be concise.
3. Your earlier reply regarding selection of three species i.e. Rhizoscyphus ericae, Hygrophorus camarophyllus, and Laccaria laccata AT2001038 is bit confusing. As you mentioned that your focus was on ECM fungal species because lots of work has been already done on saprophytic fungi. If this is true then why you have screened a large number of saprophytic fungi in your preliminary study.
4. In table 1, 2, 6-dichlorobenzamide (BAM) composition is not mentioned anywhere. During BAM preparation you have dissolved 250 mg BAM in 1 liter water but later undissolved material was removed by filtration. This would affect concentration of BAM in prepared solution and by doing so final concentration of BAM cannot be figure out.
5. Figure 1 and 2 are still confusing. Simply we can’t predict what bar represent means biomass or growth. Similarly, on X axis unit is given for BAM but not for other two compounds. It must be symmetrical.
6. Some of the sentences needs to be corrected such as:

Line: 313 to 315

Line: 434

7. In this study, we are predicting fungal ability to degrade the recalcitrant N compound just by their growth and biomass. However, we have to assess the final concentration of N compound in growth medium after completion of the experiment. Then only we can say with certainty that how much our fungal species are effective in degrading the studied compound.
8. Species identification by morphological evidences is good but for certainty and better reliability it must be supported by molecular sequencing.
