# Supplementary material for: Survival and growth of saprotrophic and mycorrhizal fungi in recalcitrant amine, amide and ammonium containing media
Source: PLoS One. 2021 Sep 1;16(9):e0244910. doi: 10.1371/journal.pone.0244910 (PMC8409640; doi:10.1371/journal.pone.0244910)
Supplement: Attachment — Submitted filename: Response to reviewers.docx [file pone.0244910.s010.docx]

**Response to reveiwers**

Please see below the final changes made to the manuscript. Our responses are stated in blue.

Reviewer #2: In this revised version, the authors have taken into consideration all the suggestions raised in my previous review and modified the manuscript accordingly. Nevertheless, there are a few minor changes necessary as mentioned below.

Line 74: Change ‘Ectomycorrhizal fungi’ to ‘Ectomycorrhizal fungi (ECM),’.

Done.

Lines 201, 226: Use the ‘degree symbol’.

Done.

Line 211: Subscript the numbers in the chemical formula.

Done.

Lines 129, 199, 288, 305, 322, 333-334, 386: It is not clear why the abbreviation for 2,6-dichlorobenzamide is introduced so many times as it is already there in Line 31.

Yes, thank you for pointing that out. We kept the introduction of compound and abbreviation in the Introduction, line 129 which is the first place it is mentioned in the main body of the text. All other places have been replaced with BAM.

Lines 347, 359–362: Express these huge percentage values in folds.

Done.

Line 372: Change ‘that is appears’ as ‘that it appears’.

Done.

Line 433: Replace ‘[5, 8]’ with ‘[5, 8],’.

Done.

Line 434: Correct the spelling for ‘to’.

Done.

Attached letter: Although authors has improved the manuscript markedly but still I have some queries and doubt regarding my earlier comments.

What is the history of sampling site? I mean fungal species isolated from any waste water disposal site would be more adaptable for degrading the recalcitrant compound compared to fungi isolated from healthy site.

Fungal collection was made in forests and other natural habitats for the sake of isolating the fungal species in their natural environment. We had an ecological angle into this study. If our aim had been to target polluted sites this would have been done differently, yes.

Introduction needs to be concise.

The Introduction was revised the first round, shortened, and focused according to suggestions. We are not sure what part of the Introduction you consider unconcise. Therefore, it is difficult to make more changes.

Your earlier reply regarding selection of three species i.e. Rhizoscyphus ericae, Hygrophorus camarophyllus, and Laccaria laccata AT2001038 is bit confusing. As you mentioned that your focus was on ECM fungal species because lots of work has been already done on saprophytic fungi. If this is true then why you have screened a large number of saprophytic fungi in your preliminary study.

The way into this study was dual: firstly, we have a purely ecological focus in that we wanted to screen relatively broadly across a number of fungal species that are commonly found in European forests. In this way we produce data that can be put into both an ecological and taxonomical context, since knowledge is limited. Although lots of work has been done on saprotrophic fungi, this work has mostly focused on very few saprotrophic species. Secondly, we wanted to focus partly on mycorrhizal fungi since they are not normally included in this type of studies, despite having similar enzyme systems as the saprotrophic fungi.

In table 1, 2, 6-dichlorobenzamide (BAM) composition is not mentioned anywhere. During BAM preparation you have dissolved 250 mg BAM in 1 liter water but later undissolved material was removed by filtration. This would affect concentration of BAM in prepared solution and by doing so final concentration of BAM cannot be figure out.

BAM was not included in the simulated process water experiment, hence it is not mentioned in Table 1.

Figure 1 and 2 are still confusing. Simply we can’t predict what bar represent means biomass or growth. Similarly, on X axis unit is given for BAM but not for other two compounds. It must be symmetrical.

In the figure legend it is already clearly stated that:

‘…Bars show biomass (mg), lines show growth as a percentage of the growth controls.’

This should be enough to explain the figure. We corrected the figures and removed the units for BAM. The information is now only included in the figure legend.

Some of the sentences needs to be corrected such as:

Line: 313 to 315

Changed to: Negative growth responses to all treatments were exemplified by (e) *Ganoderma applanatum* (brown rot) and (f) *Lenzites betula* (white rot), as well as two parasites (g) *Phanerochaete sordida*, and (h) *Rhizina undulata*.

Line: 434

In this study, we are predicting fungal ability to degrade the recalcitrant N compound just by their growth and biomass. However, we have to assess the final concentration of N compound in growth medium after completion of the experiment. Then only we can say with certainty that how much our fungal species are effective in degrading the studied compound.

We assume you wanted something along those lines appended to the discussion? We already have information about this issue towards the end of the Discussion, and in the final summery paragraph. We now added the following to the end of the starting Discussion paragraph, line 440 onwards:

‘…. In this study, we are predicting fungal ability to degrade the recalcitrant N compound by their growth and biomass. However, this first screening study needs to be followed by more in-depth studies confirming decreased concentrations of these substances.’

Species identification by morphological evidences is good but for certainty and better reliability it must be supported by molecular sequencing.

Here we need to agree to disagree, application of molecular tools is not always the most reliable way to identify fungal species. As an example the NCBI database is commonly used for identifying environmental sequences from soil amplicons, field samples etc. Unfortunately, there are many wrongly named sequences in that database (even things that are not truly fungal are stated as fungal), and one would need to use more curated information in e.g. UNITE (where entries have been confirmed by mycologists specialising in different parts of fungal taxonomy, then produced sequences from fungal fruitbody material which is correctly labelled from the beginning). When working with environmental sequences they can still be grouped into species hypothesis etc and compared top other thus grouped sequences, but we do believe that the faith in molecular tools over classical mycology is a bit inflated. In the case of the species included in the present study there are no ambiguous species that we were not sure of. The persons responsible for collecting, identifying and isolating the species are highly competent in the area of classical mycology *as well as* molecular ecology/fungal community ecology by molecular methods. We are confident that the identifications were done correctly. Finally, the study was conducted some years ago and we do not intend sequence the isolates at this point. We hope this is acceptable.
